# Targeted Isolation of a Cytotoxic Cyclic Hexadepsipeptide from the Mesophotic Zone Sponge-Associated Fungus *Cymostachys* sp. NBUF082

**DOI:** 10.3390/md19100565

**Published:** 2021-10-11

**Authors:** Ye Yuan, Te Li, Tingting Wang, C. Benjamin Naman, Jing Ye, Xingxin Wu, J. Enrico H. Lazaro, Xiaojun Yan, Shan He

**Affiliations:** 1 Department of Marine Pharmacy, Li Dak Sum Marine Biopharmaceutical Research Center, College of Food and Pharmaceutical Sciences, Ningbo University, Ningbo 315832, China; 23yuanye@163.com (Y.Y.); telinbu@163.com (T.L.); bnaman@nbu.edu.cn (C.B.N.); 2 School of Marine Science, Ningbo University, Ningbo 315832, China; 3 State Key Laboratory of Pharmaceutical Biotechnology, School of Life Sciences, Nanjing University, Nanjing 210023, China; DG1930079@smail.nju.edu.cn (J.Y.); xingxin.wu@nju.edu.cn (X.W.); 4 National Institute of Molecular Biology and Biotechnology, University of the Philippines Diliman, Quezon 1101, Philippines; jaylazaro@mbb.upd.edu.ph

**Keywords:** mesophotic zone, sponges, sponge-associated fungi, hexadepsipeptide, cytotoxicity

## Abstract

LC-MS/MS-based molecular networking facilitated the targeted isolation of a new cyclic hexadepsipeptide, cymodepsipeptide (**1**), and two known analogues, RF–2691A (**2**) and RF–2691B (**3**), from the fungus *Cymostachys* sp. NBUF082 that was derived from a mesophotic zone *Aaptos* sponge collected near Apo Island. The constitution and configuration of **1** was elucidated through 1D and 2D NMR-spectroscopy, high resolution mass-spectrometry, and chemical degradations including Marfey’s analysis and chiral HPLC. It was observed that **1** was moderately cytotoxic against CCRF-CEM human acute lymphocytic leukemia cells in vitro with the IC_50_ value of 9.2 ± 1.1 μM.

## 1. Introduction

Research on marine organisms, and especially sponge-derived microorganisms, has afforded a prolific library of new and interesting natural products for drug discovery [1,2,3,4]. From such marine biodiversity, the global marine pharmaceuticals pipeline website (https://www.marinepharmacology.org/) lists more than 15 approved drugs and over 30 drug candidates in Phase I, II, and III of clinical trials [1]. An enormous variety of compounds with complex structures and pronounced bioactivities has been reported from sponge-associated fungi, as exemplified in recent years by the chromone aspergilluone A with antimicrobial activity [5], meroterpenoids eupenicilazaphilones A–C and eupenicillin A with cytotoxic activity [6], isoprenylated cyclohexanol derivatives truncateols O–V with antiviral activity [7], polyketides eurobenzophenones A–C, euroxanthones A–B, and (+)-1-*O*-demethylvariecolorquinones A with antioxidant and NO inhibitory activities [8].

Cyclic depsipeptides are a family of peptides containing at least one or two ester bonds in addition to typical amide bonds [9]. Depsipeptides have been found in macroorganisms, including sponges [10], ascidians [11] and lichens [12], and microorganisms, such as cyanobacteria [13], bacteria [14], and fungi [15]. This chemical class has emerged as a significant source of lead compounds for pharmaceutical research due to a wide array of associated bioactivities, including anti-hypertensive [16], cytotoxic [17], anti-bacterial [18], and antiviral activities [19] among others. More focused efforts to discover new depsipeptides is expected to be productive and beneficial in natural product drug discovery programs.

**Figure 1:**
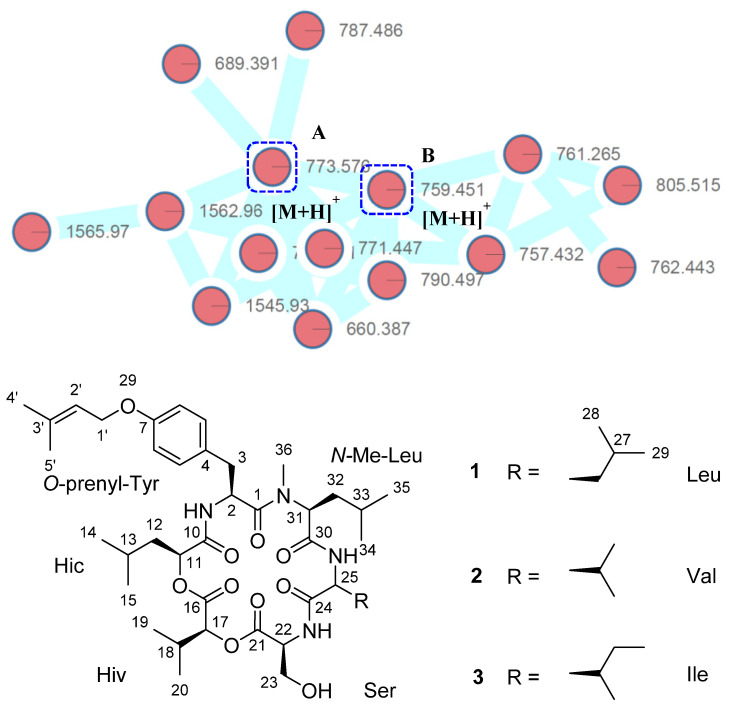
The structure panel of compounds **1**–**3** beneath the molecular networking cluster that contains nodes corresponding to **1** and **3** (Node A) and **2** (Node B). This cluster was extracted from the whole molecular network of the crude extract (Figure S1) for visualization purposes.

Mesophotic coral ecosystems (MCEs, 30 to 150 m deep) harbor tremendous biodiversity, even some 80% of coral reef habitat globally, but literature reports on MCEs remain rare compared to those about shallow reefs [20]. In ongoing efforts to discover interesting natural products from fungi in this frontier environment, the *Aaptos* sponge-associated fungus *Cymostachys* sp. NBUF082 was previously reported to produce novel low-order polymers of a new aromatic polyol monomer, 7-methoxy-1,3-dihydroisobenzofuran-5-ol, and these have anti-*Vibrio* activities [21]. Further analysis of extract of this fungus by LC-MS/MS based molecular networking allowed for a targeted strategy to isolate a set of structurally related compounds from other chemical classes. From this study, a new cytotoxic cyclic hexadepsipeptide, cymodepsipeptide (**1**), and two known analogues, RF–2691A (**2**) and RF–2691B (**3**) (Figure 1) [22], were obtained. Herein described are the targeted isolation, structure elucidation, and biological evaluation of these compounds.

## 2. Results and Discussion

The crude extract of *Cymostachys* sp. NBUF082 was subjected to LC-MS/MS analysis, and a molecular network was generated using the Global Natural Product Social (GNPS) Molecular Networking platform (Figure S1). From the total molecular network, a cluster of nodes was found to represent a set of related metabolites from this strain that had similar MS/MS fragmentation patterns that strongly suggested a polypeptidic core scaffold (Figure 1). Several of these nodes were able to be correlated with typical mass adducts, for example parent masses observed at *m/z* 776 and 790 appear to be ammonium adducts of **1**–**3**. The node with parent mass *m/z* 660, based on the chromatographic retention time and MS/MS fragmentation pattern, seems to be a mutually shared source fragmentation of **1**–**3**. The mass difference between the isolated compounds and this fragment indicates the removal of a Val, *N*-Me Val, Leu or Ile residue. Other nodes observed included in-source dimers with different adducts, such as *m/z* 1545 and 1562. Remaining nodes corresponded with ions that were at significantly reduced intensity (≤1%) compared to those of the isolated molecules.

Compound **1** was isolated as a white powder and determined to have the molecular formula of C_41_H_64_N_4_O_10_ based on a sodium adduct peak in the high resolution electrospray ionization mass spectrometry (HRESIMS) at *m/z* 795. 4506 [M + Na]^+^ (calcd. for C_41_H_64_N_4_O_10_Na, 795.4515), indicating 12 degrees of unsaturation. The NMR data of **1** showed the co-existence of five pairs of vicinal methyls, an *N*-methyl group, six methylenes of which two are oxygenated, five olefinic protons including four aromatics, ten methines involving six peptidic *α*-methines, three amide protons, six ester and/or amide carbonyl carbons, together with three sp^2^ non-protonated carbons (Table 1). The tabulated functionalities accounted for 11 degrees of unsaturation, suggesting that **1** could be macrocyclic to fulfil the unsaturation requirement of the molecular formula. Since the molecular formula indicated only four nitrogen atoms, while the NMR data presented six peptidic *α*-methine protons, it was suggested that **1** may belong to the cyclodepsipeptide family and contain two ester bonds resulting from the presence of α-hydroxy acids in the place of α-amino acids.

**Figure 2:**
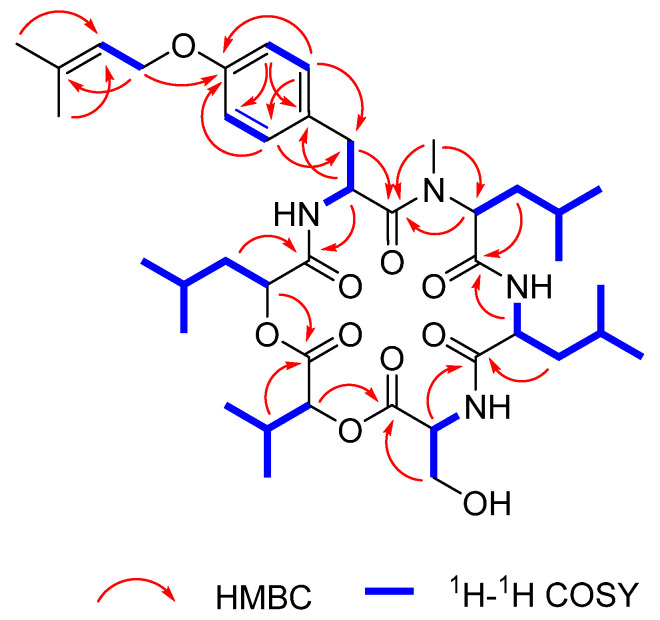
Selected HMBC and ^1^H-^1^H COSY correlations for **1**.

^1^H-^1^H COSY correlations with amide NH and *α*-methine protons were used to establish a series of *α*-hydroxy and *α*-amino acid residues from the NMR data. A hydroxyisocaproic acid (Hic) moiety, a hydroxyisovaleric acid (Hiv) moiety, a serine (Ser) moiety, a leucine (Leu) moiety, and the second Leu moiety were clearly distinguished from the coupling systems demonstrated in the ^1^H-^1^H COSY spectrum (Figure 2). Cross-peaks in the HMBC spectrum of **1**, from *N*-methyl group CH_3_-36 (*δ*_H_ 3.10/ *δ*_C_ 41.0) to C-31 indicated that the second Leu substructure was an *N*-Me-Leu residue. Furthermore, four spin systems starting from the amide proton at *δ*_H_ 7.47~H-2‒H-3, H-5‒H-6, H-8‒H-9, and H-1′‒H-2′ revealed by ^1^H-^1^H COSY data were all interconnected by HMBC correlations of H-2 to C-4, H-5/H-9 to C-3, H-5 to C-7/C-9, H-6 to C-4/C-8, H-9 to C-7, H-1’ to C-7/C-3’, and H_3_-4′/H_3_-5′ to C-2′ to assign an *O*-prenyl-Tyr motif. The crosspeaks of H-3/H_3_-36 to C-1, H-2/H-12 to C-10, H-11/H-18 to C-16, H-17/H-23 to C-21, H-22/H-26 to C-24, and H-25/H-32 to C-30 in the HMBC spectrum were used to conclusively elucidate the planar structure of **1**, a new cyclic hexadepsipeptide. 

A stereochemical study of **1** was completed after hydrolysis by using Marfey’s method for the *α*-amino acids [23] and chiral HPLC analysis for the *α*-hydroxy acids [24]. The Marfey’s analysis using Nalpha-(5-Fluoro-2,4-dinitrophenyl)-d-leucinamide (d-FDLA) revealed that the Ser, Leu, *O*-prenyl-Tyr and *N*-Me-Leu residues were all of l-configuration. The chiral HPLC analysis of the diethyl ether extract of the hydrolysate, along with authentic *S*- and *R*- Hic/Hiv standards showed that the Hic and Hiv moieties of **1** are both *S*. 

Compounds **2** and **3** were determined to be RF–2691A (**2**) and RF–2691B (**3**) by comparisons of the experimentally observed MS, NMR and optical rotation data values with those reported in the literature [22]. Compounds **1** and **3** have the same molecular weight and differ in constitution by a single residue, alternatively Leu and Ile. These compounds were separated chromatographically by HPLC but, because they have identical MS/MS spectra, GNPS combined them into a single consensus node, visualized in Figure 1 as Node A.

Quite a few cyclic depsipeptides are known for their cytotoxicity [15,24,25,26], thus compound **1** was screened in vitro against human T lymphoblast cells CCRF-CEM human acute lymphocytic leukemia cells. The results showed that compound **1** exhibited moderate inhibition towards CCRF-CEM cells with the IC_50_ value of 9.2 ± 1.1 μM. Compounds **2** and **3** did not show activity towards CCRF-CEM cells at the concentration of 20.0 μM in preliminary screening together with compound **1** but were insufficient to support further bioassay for their IC_50_ values. However, compounds **1**–**3** were found to be inactive at concentrations as high as 100.0 μM on U87 cells, perhaps due to solubility issues or sample aggregation. 

## 3. Materials and Methods

### 3.1. General Experimental Procedures

NMR spectra were tested on Bruker AVANCE NEO 600 spectrometer with a 5 mm inverse detection triple resonance (H-C/N/D) cryoprobe with z-gradients. Optical rotations were recorded on an SGW-532 automatic polarimeter in MeOH at 20 °C. NMR spectra were tested on Bruker AVANCE NEO 600 spectrometer with a 5 mm inverse detection triple resonance (H-C/N/D) cryoprobe with z-gradients. All spectra were measured with standard Bruker pulse programs, and ^1^H and ^13^C chemical shifts were expressed on the solvent CDCl_3_ (*δ*_H_ 7.26 and *δ*_C_ 77.00). A normal phase column was performed with silica gel (200–300 mesh) from Qingdao Marine Chemical Company, China. Sephadex LH-20 (Pharmacia Biotech, Uppsala, Sweden) was used for gel filtration, and ODS-A GEL (AA12S50; YMC Co., Ltd., Kyoto, Japan) for reverse phase column chromatography. High-resolution electrospray ionization (HRESIMS) spectra were determined on an Agilent 6545 Q-TOF instrument (Santa Clara, CA, USA). Semi-preparative HPLC separation was carried out on reversed-phase HPLC; purification was performed using an ODS-2 Hypersil column (5 µm, 250 × 10 mm) on a Waters liquid chromatography system equipped with a 1525 binary pump and a W2998 diode array detector (Waters Corporation, Milford, MA, USA). Separations of extracts and sub-fractions were monitored by thin-layer chromatography (GF254, 10–20 μm), and compounds were visualized by heating silica gel plates coated with 20% H_2_SO_4_ in EtOH. Chiral HPLC analysis was performed on a Chirex 3126 (d)-penicillamine (5 μm; 250 × 4.6 mm) (Phenomenex, Torrance, CA, USA). The isolation and characterization of the fungus *Cymostachys* sp. NBUF082 researched in this work has been described previously [21]. 

### 3.2. Cultivation and Extraction

The stain was cultivated in 324 × 1 L Erlenmeyer flasks containing 400 mL PDB medium (80 g potato dextrose, 8 g glucose, 14.0 g sea salt, and 400 mL H_2_O) followed by 16-day fermentation at 28 °C (120 rpm) for 129.6-L scale. The whole cultures were filtered through cheese cloth into filtrate and mycelia. The filtrate was extracted with EtOAc (1:1, *v*/*v*) 3 times, while the mycelia were treated with DCM−MeOH (1:1, *v*/*v*) 5 times. The obtained two EtOAc extract liquors were concentrated under the reduced pressure and combined based on the TLC analysis to afford the final residue (50 g).

### 3.3. Molecular Networking and Isolation of ***1***

A sample of the crude extract was filtered through a 0.22 μm membrane and dissolved in MeOH with the concentrations of 1 mg/mL. An amount of 3 μL of the obtained liquor was injected into the LC-HRESIMS and eluted at 0.8 mL/min (MeOH/H_2_O with 0.1% formic acid, *v*/*v*, 30%→99%): 30% for 5 min to 99% in 17 min, held for 3 min, to 30% in 1 min, and held for 4 min. The mass spectrometer was set to monitor *m/z* 190–2000 in positive ESI mode and enable an automated data-dependent MS/MS scan. The generated data were uploaded to the Global Natural Product Social Molecular Networking web interface (GNPS). The processed results were used to visualize the molecular network with the open source software Cytoscape. 

The obtained crude extract was subjected to chromatography (CC) over silica gel (PE/EtOAc, *v*/*v*, 100:0→0:100 then EtOAc/MeOH *v*/*v*, 100:0→0:100) to give 9 fractions (Fr.1–Fr.9). Further separation of Fr.6 with Sephadex LH-20 in MeOH yielded 10 subfractions (Fr.6.1–Fr.6.10). Fr.6.2 was purified via RP-HPLC (CH_3_CN/H_2_O, 65:35) to afford **1** (*t*_R_ = 45 min, 8.6 mg), **2** (*t*_R_ = 42 min, 2.4 mg), and **3** (*t*_R_ = 48 min, 1.4 mg). The isolation yield of compounds **1**–**3** from crude extract were approximately 0.017%, 0.005% and 0.003%, respectively. 

### 3.4. Physio–Chemical Data of **1**

Cymodepsipeptide (**1**): white powder; [*α*${]}_{D}^{20}$ = −64.1° (*c* = 0.1, MeOH); UV (MeOH), *λ*_max_ (log *ε*) = 205 nm (2.89); for ^1^H and ^13^C NMR data see Table 1; HR-ESI-MS [M + Na]^+^ *m/z* 795. 4506 (calcd. for C_41_H_64_N_4_O_10_Na^+^, 795.4515).

### 3.5. Acid Hydrolysis, Amino Acid Analysis of Marfey’s Derivatives of ***1***

Compound **1** (1.0 mg) dissolved in acetone (30 μL) was hydrolyzed in 6 N HCl (1.0 mL) at 115 °C for 24 h. The mixture was dried after cooling. The residue and amino acid standards (1.0 mg each) were re-dissolved in H_2_O (100 μL), with 1% d-FDLA (200 μL) and 1 M NaHCO_3_ (40 μL) added later. The reaction system was stirred at 45 °C for 1 h, and then acidized with 2 N HCl (20 μL). The final mixture was dried, filtered and subjected to LC-MS analysis with solvent system [CH_3_CN (0.1% formic acid): H_2_O (0.1% formic acid)→10–100% in 50 mins at a flow rate of 1 mL/min]. Retention times for the d-FDLA derivatized amino acid standards were as follows: d-Tyr (*t*_R_ = 18.428 min), l-Tyr (*t*_R_ = 19.753 min), d-Leu (21.031 min), l-Leu (25.742 min), d-*N*-Me-Leu (22.921 min), l-*N*-Me-Leu (24.939 min), d-Ser (14.897 min) and l-Ser (15.354 min). The d-FDLA-derivatized **1**-hydrolysate peaks determined by expected masses were observed at 19.770 min, 25.728 min, 24.885 min, and 15.293 min, in accord with those of l-Tyr, l-Leu, l-*N*-Me-Leu, and l-Ser. Compound **1** (2.0 mg) was hydrolyzed in the same way with above and subjected to chiral HPLC analysis (5% 2-propanol in 2 mM CuSO_4_, 1 mL/min). Retention times for the *S*- and *R*- Hic/Hiv standards were as follows: *S*-Hic (34.086 min), *R*-Hic (43.420 min), *S*-Hiv (10.562 min), *R*-Hiv (17.358 min). 

### 3.6. In Vitro Cytotoxicity Test Protocols

Compounds **1**–**3** were evaluated for cytotoxicity against human T lymphoblast cells CCRF-CEM and human glioblastoma U87 with MTT according to protocols reported elsewhere [27]. In brief, 180 μL suspended cells were seeded into each well of 96-well cell culture plates and inoculated at 37 °C in a 5% CO_2_ chamber before test samples addition. Compounds **1**–**3** dissolved in DMSO at the working concentration of 20.0, 10.0, 5.0, 2.5, 1.25 μM were added into the cell-containing wells, with Doxorubicin and blank medium used as positive and negative controls. The tumor cells exposed to test compounds and Doxorubicin were stained by MTT (thiazolyl blue tetrazolium bromide 98%; Sigma-Aldrich, St. Louis, MO, USA), then the optical density of the lysate was measured at 490 and 570 nm on a ThermoElectron Multiskan Ascent plate reader (Thermo, Waltham, MA, USA). The IC_50_ value of test compounds was evaluated by Reed and Muench’s method.

## 4. Conclusions

In summary, one new cyclic hexadepsipeptide cymodepsipeptide (**1**), and the two known analogues, RF–2691A (**2**) and RF–2691B (**3**), were characterized from the *Cymostachys* sp. NBUF082 fungus associated with a mesophotic zone *Aaptos* sp. sponge, after re-mining LC-MS/MS molecular networking data. Cymodepsipeptide (**1**) showed cytotoxicity towards CCRF-CEM cells and indicates a meaningful bioactivity for the cyclohexadepsipeptide chemotype. The present yield rate of **1**–**3** was estimated as 0.017%, 0.005% and 0.003%, respectively, the relatively low obtainment of targeted compounds may be due to relatively low expression of genes for polypeptides in current culture conditions. Notably, the molecular networking cluster that contained the compounds targeted indicated more cyclic hexadepsipeptides in addition to **1**–**3**, which may have been below isolable quantities but could remain to be discovered in a scaled-up sample production and regulation of peptide gene expression by nutrients and genetic engineering technology. Sponge-associated fungi in MCEs represent prolific producers of natural products, and the incorporation of modern mining strategies with traditional natural product discovery is expected to yield intriguing structures with pronounced bioactivities in for drug development. 

## Figures and Tables

**Table 1:** ^1^H NMR (600 MHz) and ^13^C NMR (150 MHz) data of compound **1** in CDCl_3_.

Position	*δ*_C_	*δ*_H_, Mult. (*J* in Hz)	Position	*δ*_C_	*δ*_H_, Mult. (*J* in Hz)
*O*-prenyl-Tyr			Hiv		
1	172.6		16	167.8	
2	50.2	5.27 ddd (10.3, 8.6, 4.7)	17	79.1	4.67 d (5.0)
3	38.6	2.81 dd (13.3, 10.3) 2.94 dd (13.3, 4.7)	18	30.4	2.28 m
4	127.7		19	17.6	1.05 d (6.8)
5	130.3	7.12 d (8.6)	20	18.8	1.04 d (6.8)
6	114.6	6.82 d (8.6)	Ser		
7	158.0		21	170.2	
8	114.6	6.82 d (8.6)	22	55.0	4.76 td (2.1, 8.1)
9	130.4	7.12 d (8.6)	23	63.4	4.08 dd (12.2, 2.1) 4.23 dd (12.2, 2.1)
1’	64.7	4.46 d (6.8)	NH Leu		7.95 d (8.1)
	
2’	119.5	5.46 m	24	171.9	
3’	138.4		25	51.8	4.55 m
4’	25.8	1.80 s	26	39.1	1.55 m, 1.96 m
5’	18.2	1.74 s	27	25.1	1.62 m
NH		7.47 d (8.6)	28	21.4	0.90 d (6.6)
Hic			29	23.3	0.97 d (6.6)
10	169.7		NH *N*-Me-Leu		5.95 d (8.7)

11	73.5	5.47 dd (8.2, 4.7)	30	170.5	
12	42.1	1.58 m, 1.72 m	31	65.2	3.50 dd (9.0, 6.2)
13	24.6	1.63 m	32	36.9	1.37 m, 1.71 m
14	23.0	0.94 d (6.3)	33	24.5	1.02 m
15	22.0	0.93 d (6.3)	34	21.7	0.85 d (6.5)
			35	23.5	0.83 d (6.5)
			36	41.0	3.10 s

## Supplementary Materials

The following are available online at https://www.mdpi.com/article/10.3390/md19100565/s1, LC-MS/MS-derived molecular network of extracts from the fungus *Cymostachys* sp. NBUF082; ^1^H NMR, ^13^C NMR, DEPT135, ^1^H-^1^H COSY, HSQC, and HMBC spectra for compound **1**; amino acid analysis of Marfey’s derivatives, acid hydrolysis of **1**.

## References

[1] Mayer A.M.S., Rodríguez A.D., Taglialatela-Scafati O., Fusetani N. (2017). Marine pharmacology in 2012–2013: Marine compounds with antibacterial, antidiabetic, antifungal, anti-inflammatory, antiprotozoal, antituberculosis, and antiviral activities. Mar. Drugs.

[2] Wang W.Y., Yang J., Liao Y.Y., Cheng G., Chen J., Mo S.W., Yuan L., Cheng X.D., Qin J.J., Shao Z.Z. (2020). Aspeterreurone A, a cytotoxic dihydrobenzofuran–phenyl acrylate hybrid from the deep-sea-derived fungus *Aspergillus terreus* CC-S06-18. J. Nat. Prod..

[3] Sahidin I., Sabandar C.W., Wahyuni, Hamsidi R., Mardikasari S.A., Zubaydah W.O.S., Sadarun B., Musnina W.O.S., Darmawan A., Sundowo A. (2020). Investigation of compounds and biological activity of selected Indonesian marine sponges. Nat. Prod. J..

[4] Rangnekar S., Khan T. (2015). Novel anti-inflammatory drugs from marine microbes. Nat. Prod. J..

[5] Liu Y., Ding L., He J., Zhang Z., Deng Y., He S., Yan X. (2021). New antibacterial chromone from a marine sponge-associated fungus *Aspergillus* sp. LS57. Fitoterapia.

[6] Gu B.B., Wu Y., Tang J., Jiao W.H., Li L., Sun F., Wang S.P., Yang F., Lin H.W. (2018). Azaphilone and isocoumarin derivatives from the sponge-derived fungus *Eupenicillium* sp. 6A-9. Tetrahedron Lett..

[7] Zhao Y., Liu D., Proksch P., Zhou D., Lin W. (2018). Truncateols O-V, further isoprenylated cyclohexanols from the sponge-associated fungus *Truncatella angustata* with antiviral activities. Phytochemistry.

[8] Du X., Liu D., Huang J., Zhang C., Proksch P., Lin W. (2018). Polyketide derivatives from the sponge associated fungus *Aspergillus europaeus* with antioxidant and NO inhibitory activities. Fitoterapia.

[9] Stawikowski M., Cudic P. (2007). Depsipeptide synthesis. Methods Mol. Biol..

[10] Prasad P., Aalbersberg W., Feussner K.D., Van Wagoner R.M. (2011). Papuamides E and F, cytotoxic depsipeptides from the marine sponge *Melophlus* sp.. Tetrahedron.

[11] Machida K., Arai D., Katsumata R., Otsuka S., Yamashita J.K., Ye T., Tang S., Fusetani N., Nakao Y. (2018). Sameuramide A, a new cyclic depsipeptide isolated from an ascidian of the family Didemnidae. Bioorg. Med. Chem..

[12] Seo C., Yim J.H., Lee H.K., Park S.M., Sohn J.H., Oh H. (2008). Stereocalpin A, a bioactive cyclic depsipeptide from the Antarctic lichen *Stereocaulon alpinum*. Tetrahedron Lett..

[13] Phyo M.Y., Katermeran N.P., Goh J.X., Tan L.T. (2021). Trikoveramides A-C, cyclic depsipeptides from the marine cyanobacterium *Symploca hydnoides*. Phytochemistry.

[14] Chen Y., Liu R.H., Li T.X., Huang S.S., Kong L.Y., Yang M.H. (2017). Enduspeptides A-F, six new cyclic depsipeptides from a coal mine derived *Streptomyces* sp.. Tetrahedron.

[15] Cueto M., Jensen P.R., Fenical W. (2000). *N*-Methylsansalvamide, a cytotoxic cyclic depsipeptide from a marine fungus of the genus *Fusarium*. Phytochemistry.

[16] Meleka M.M., Edwards A.J., Xia J., Dahlen S.A., Mohanty I., Medcalf M., Aggarwal S., Moeller K.D., Mortensen O.V., Osei-Owusu P. (2019). Anti-hypertensive mechanisms of cyclic depsipeptide inhibitor ligands for G q/11 class G proteins. Pharmacol. Res..

[17] Zhou Z., Wang X., Zhang H., Sun J., Zheng L., Liu H., Wang J., Shen A., Geng M., Guo Y. (2015). Chromopeptide A, a highly cytotoxic depsipeptide from the marine sediment-derived bacterium *Chromobacterium* sp. HS-13-94. Acta Pharm. Sin. B.

[18] Chen L., Zhao W., Jiang H.L., Zhou J., Chen X.M., Lian Y.Y., Jiang H., Lin F. (2018). Rakicidins G-I, cyclic depsipeptides from marine *Micromonospora chalcea* FIM 02-523. Tetrahedron.

[19] Shin H.J., Rashid M.A., Cartner L.K., Bokesch H.R., Wilson J.A., McMahon J.B., Gustafson K.R. (2015). Stellettapeptins A and B, HIV-inhibitory cyclic depsipeptides from the marine sponge *Stelletta* sp.. Tetrahedron Lett..

[20] Pyle R.L., Copus J.M., Loya Y., Puglise K. (2019). Mesophotic coral ecosystems: Introduction and overview. Mesophotic Coral Ecosystems.

[21] Wang T., Zhou J., Zou J., Shi Y., Zhou W., Shao P., Yu T., Cui W., Li X., Wu X. (2021). Discovery of cymopolyphenols A–F from a marine mesophotic zone *Aaptos* sponge-associated fungus *Cymostachys* sp. NBUF082. Front. Microbiol..

[22] Tadashi Y., Toshuki K., Yoshimi K., Koichi M., Hiroshi I. (1994). Preparation of Acyl CoA Cholesterol Acyltransferase Inhibitor with Didymostilbe. Japanese Patent.

[23] Harada K.I., Fujii K., Hayashi K., Suzuki M., Ikai Y., Oka H. (1996). Application of D,L-FDLA derivatization to determination of absolute configuration of constituent amino acids in peptide by advanced Marfey’s method. Tetrahedron Lett..

[24] Sy-Cordero A.A., Graf T.N., Adcock A.F., Kroll D.J., Shen Q., Swanson S.M., Wani M.C., Pearce C.J., Oberlies N.H. (2011). Cyclodepsipeptides, sesquiterpenoids, and other cytotoxic metabolites from the filamentous fungus *Trichothecium* sp. (MSX 51320). J. Nat. Prod..

[25] El-Elimat T., Figueroa M., Ehrmann B.M., Cech N.B., Pearce C.J., Oberlies N.H. (2013). High-resolution MS, MS/MS, and UV database of fungal secondary metabolites as a dereplication protocol for bioactive natural products. J. Nat. Prod..

[26] Ebrahim W., Kjer J., El Amrani M., Wray V., Lin W., Ebel R., Lai D., Proksch P. (2012). Pullularins E and F, two new peptides from the endophytic fungus *Bionectria ochroleuca* isolated from the mangrove plant *Sonneratia caseolaris*. Mar. Drugs.

[27] Williams R.B., Martin S.M., Lawrence J.A., Norman V.L., O’Neil-Johnson M., Eldridge G.R., Starks C.M. (2017). Isolation and identification of the novel tubulin polymerization inhibitor bifidenone. J. Nat. Prod..

