# Supplementary material for: Targeted Isolation of a Cytotoxic Cyclic Hexadepsipeptide from the Mesophotic Zone Sponge-Associated Fungus Cymostachys sp. NBUF082
Source: Mar Drugs. 2021 Oct 11;19(10):565. doi: 10.3390/md19100565 (PMC8540034; doi:10.3390/md19100565)
Supplement: Supplementary file 1 [file marinedrugs-19-00565-s001.zip › marinedrugs-1397934-supplementary.pdf]

Supplementary Information

Targeted Isolation of a Cytotoxic Cyclic Hexadepsipeptide from the Mesophotic Zone Sponge-Associated Fungus *Cymostachys* sp. NBUF082

Ye Yuan [1,2], Te Li [1,2], Tingting Wang [1,*], C. Benjamin Naman [1], Jing Ye [3], Xingxin Wu [3], J. Enrico H. Lazaro [4], Xiaojun Yan [2,*], Shan He [1,*]

1 Department of Marine Pharmacy, Li Dak Sum Marine Biopharmaceutical Research Center, College of Food and Pharmaceutical Sciences, Ningbo University, Ningbo, Zhejiang 315832, China; 23yuanye@163.com (Y.Y.); telinbu@163.com (T.L.); bnaman@nbu.edu.cn (C.B.N.)
2 School of Marine Science, Ningbo University, Ningbo 315211, China
3 State Key Laboratory of Pharmaceutical Biotechnology, School of Life Sciences, Nanjing University, Nanjing 210023, China; DG1930079@smail.nju.edu.cn (J.Y.); xingxin.wu@nju.edu.cn (X.W.)
4 National Institute of Molecular Biology and Biotechnology, University of the Philippines Diliman, Quezon 1101, Philippines; jaylazaro@mbb.upd.edu.ph
* Correspondence: wangtingting1@nbu.edu.cn (T.W.); yanxiaojun@nbu.edu.cn (X.Y.); heshan@nbu.edu.cn (S.H.)

Table of Contents

Figure S1. LC-MS/MS-derived molecular network of extracts from the fungus *Cymostachys* sp. NBUF082. The targeted nodes correlating to cyclic hexadepsipeptides were involved in the framed cluster. 3
Figure S2. ^{1}H NMR spectrum of **1** in $CDCl_3$ (600 MHz). 4
Figure S3. ^{13}C NMR spectrum of **1** in $CDCl_3$ (150 MHz). 5
Figure S4. DEPT135 spectrum of **1** in $CDCl_3$ (150 MHz). 6
Figure S5. ^{1}H-^{1}H COSY spectrum of **1** in $CDCl_3$. 7
Figure S6. HSQC spectrum of **1** in $CDCl_3$. 8
Figure S7. HMBC spectrum of **1** in $CDCl_3$. 9
Figure S8. Chromatographic profiles of amino acids from compound **1** acid hydrolysis products 11
Figure S9. Chiral HPLC analysis performed on hydrolysis products of **1**. 12

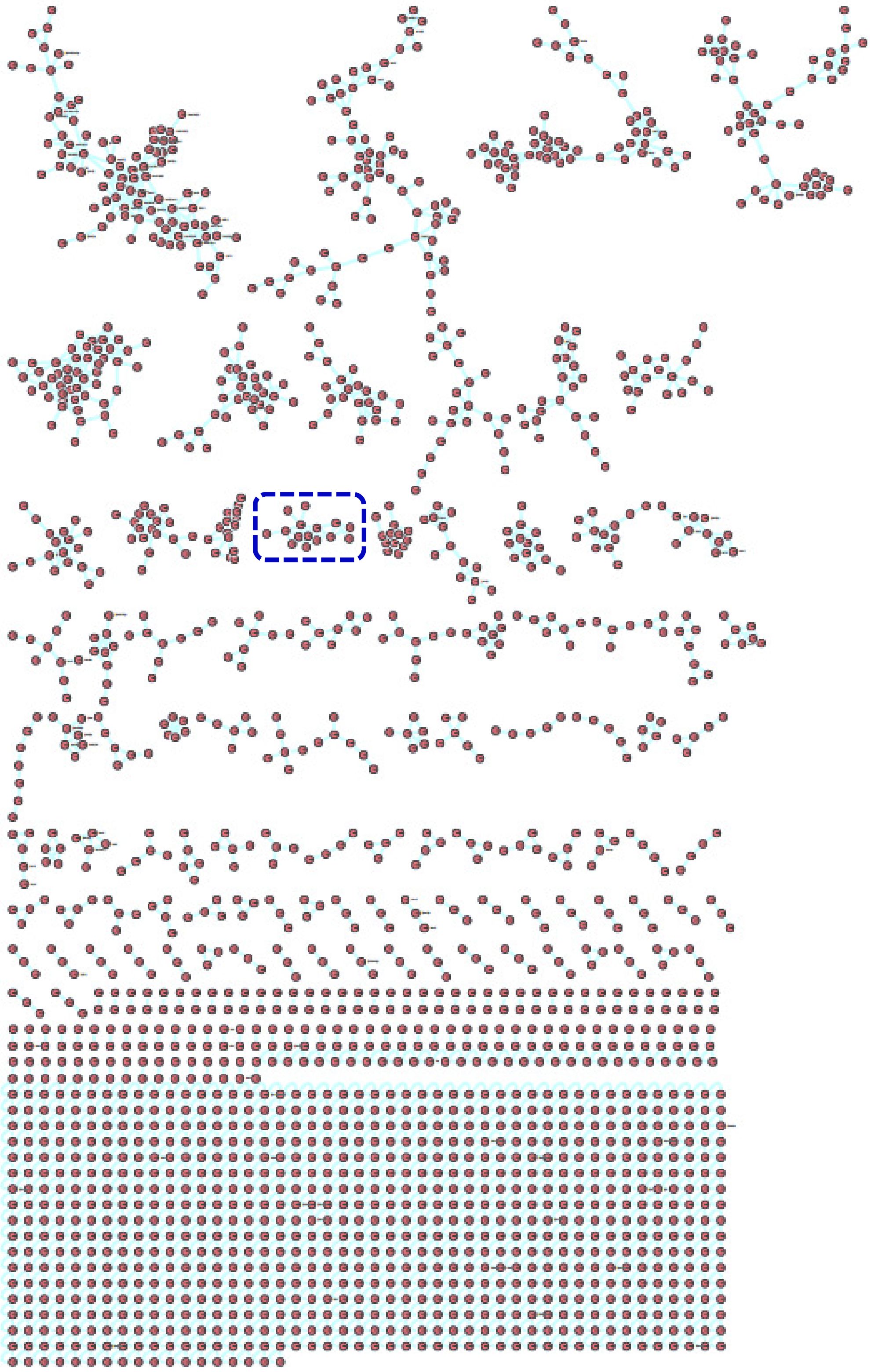

Figure S1. LC-MS/MS-derived molecular network of extracts from the fungus *Cymostachys* sp. NBUF082. The targeted nodes correlating to cyclic hexadepsipeptides were involved in the framed cluster.

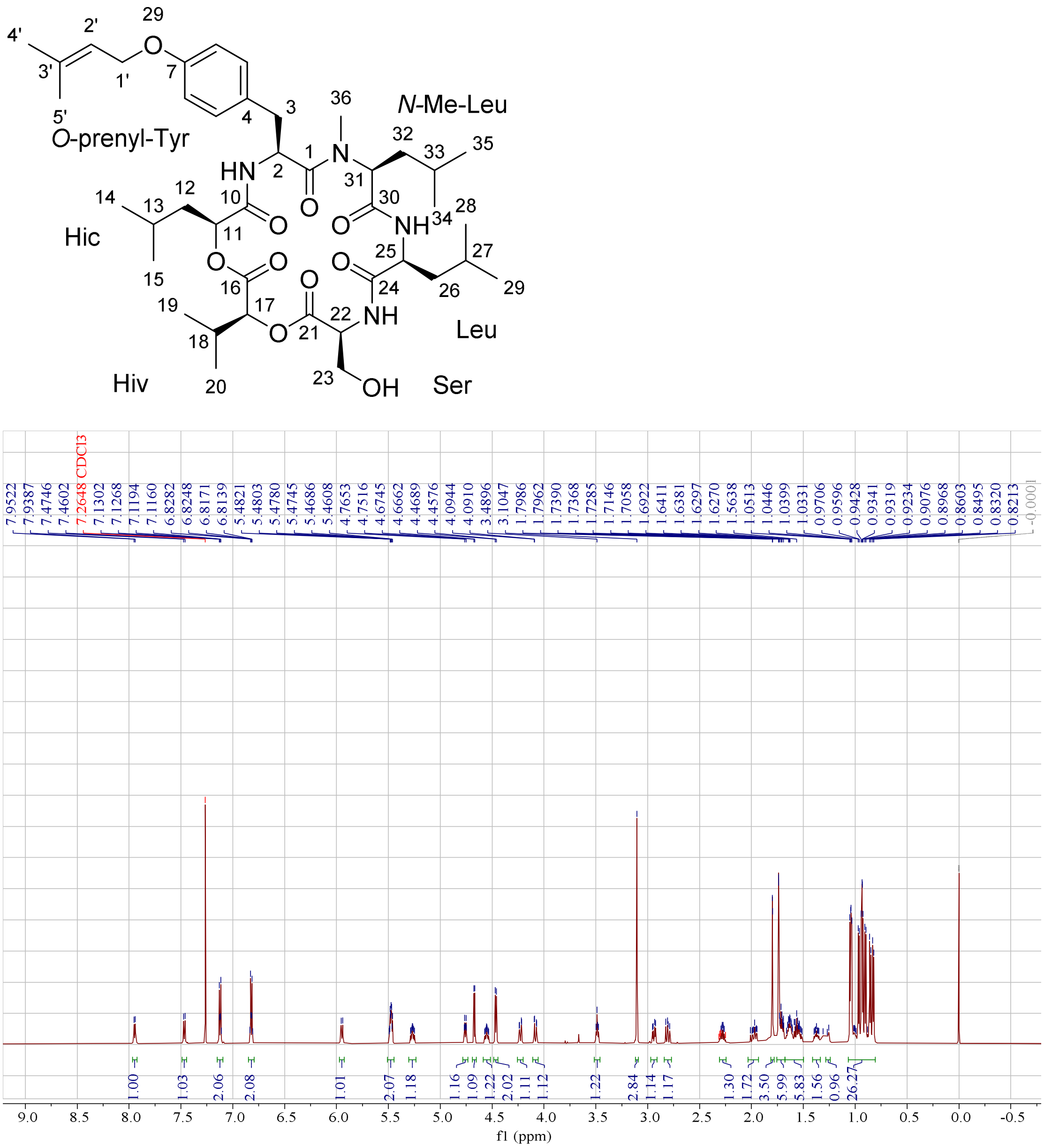

Figure S2. ^{1}H NMR spectrum of **1** in $CDCl_3$ (600 MHz).

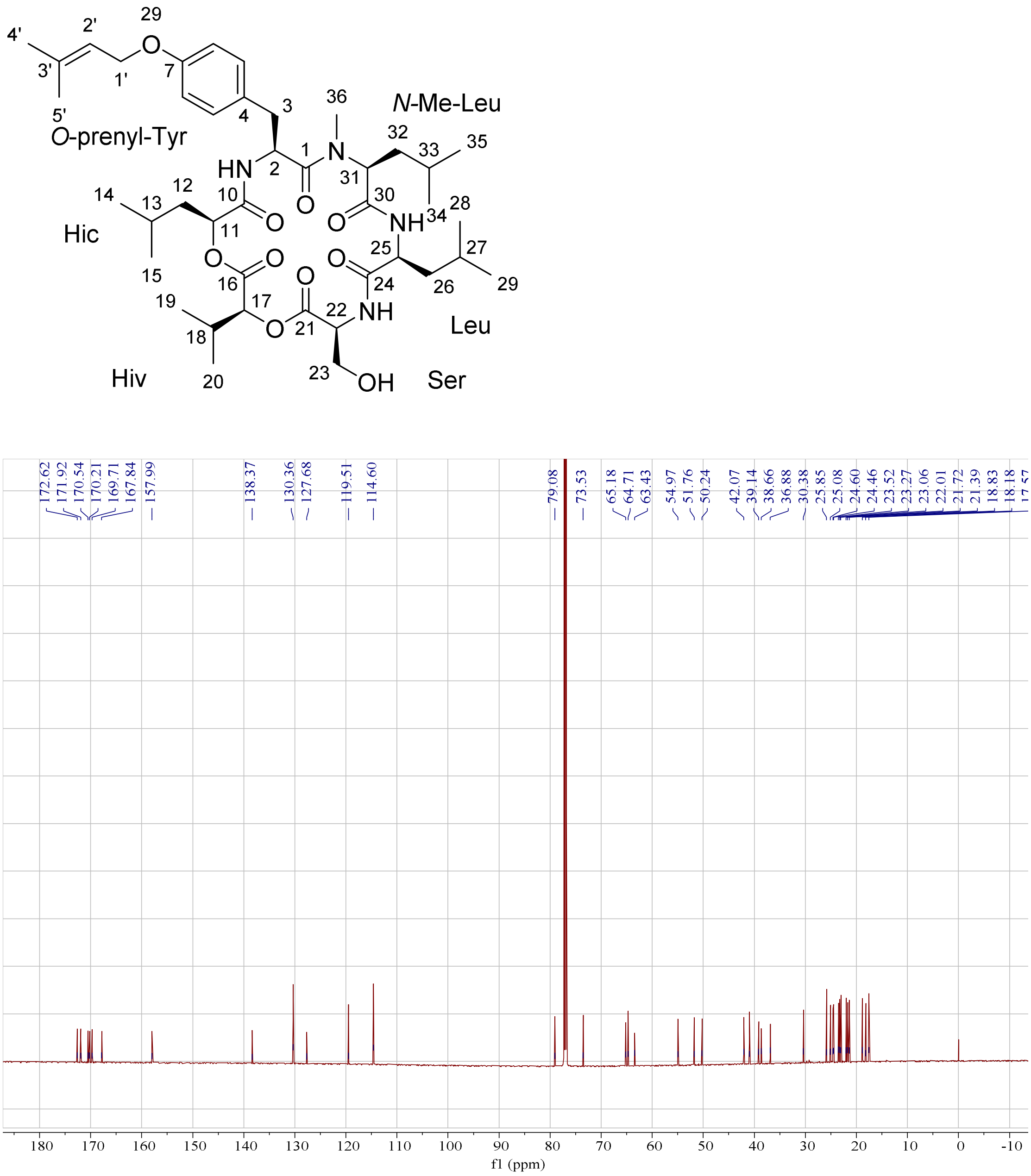

Figure S3. ^{13}C NMR spectrum of **1** in $CDCl_3$ (150 MHz).

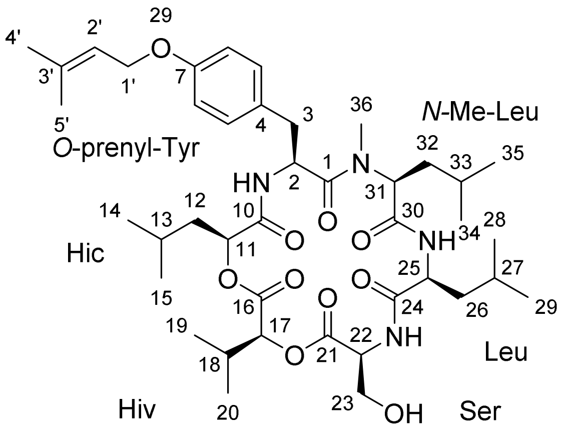

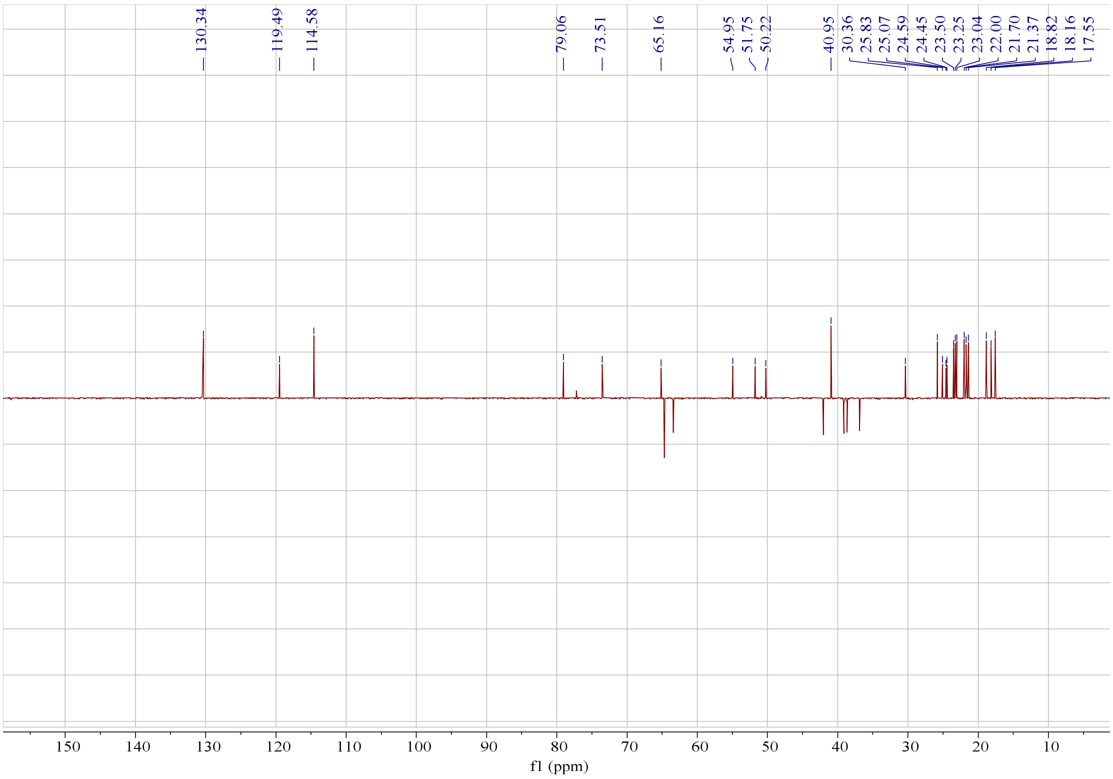

Figure S4. DEPT135 spectrum of **1** in $CDCl_3$ (150 MHz).

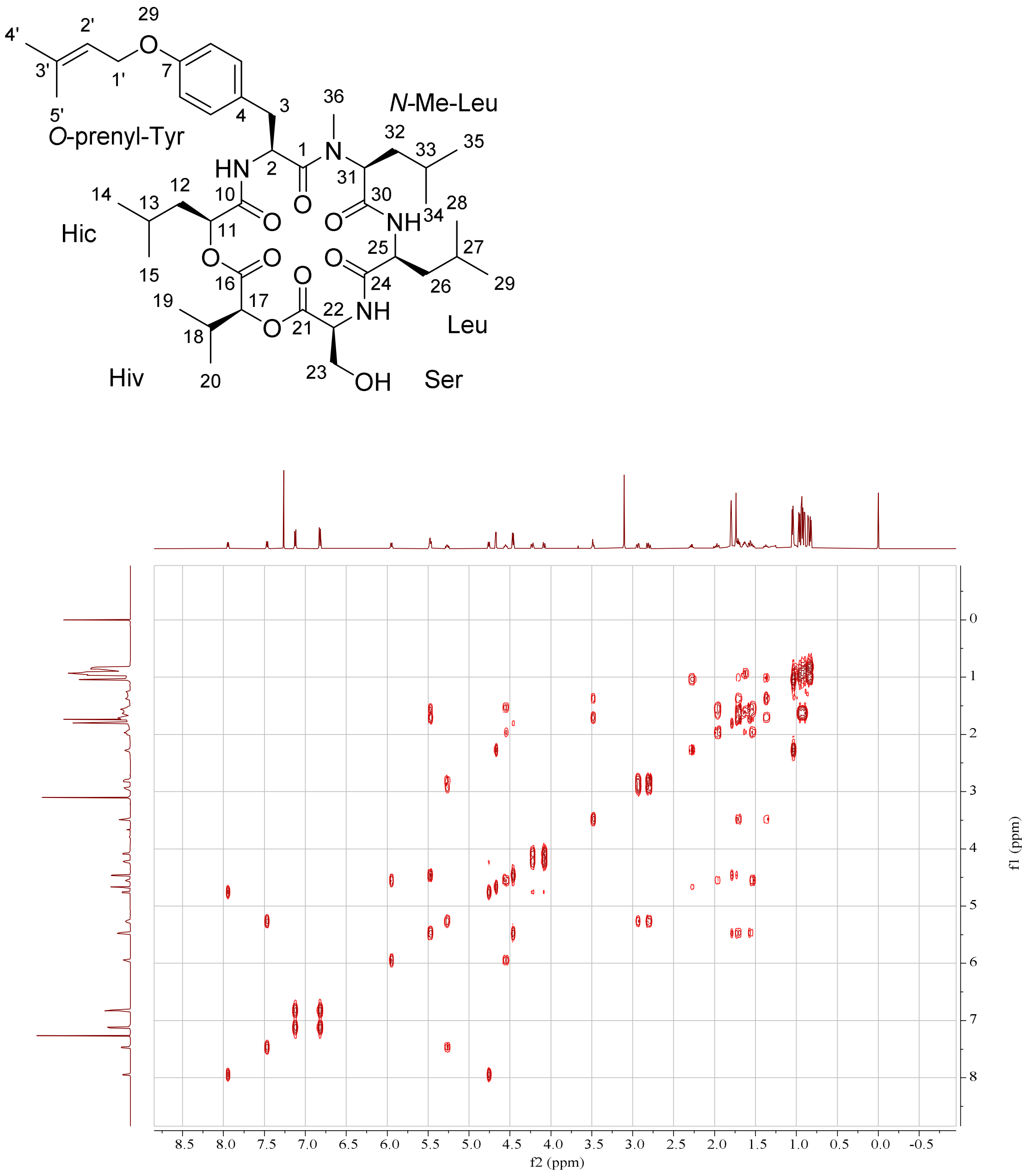

Figure S5. ^1H-^1H COSY spectrum of **1** in $CDCl_3$.

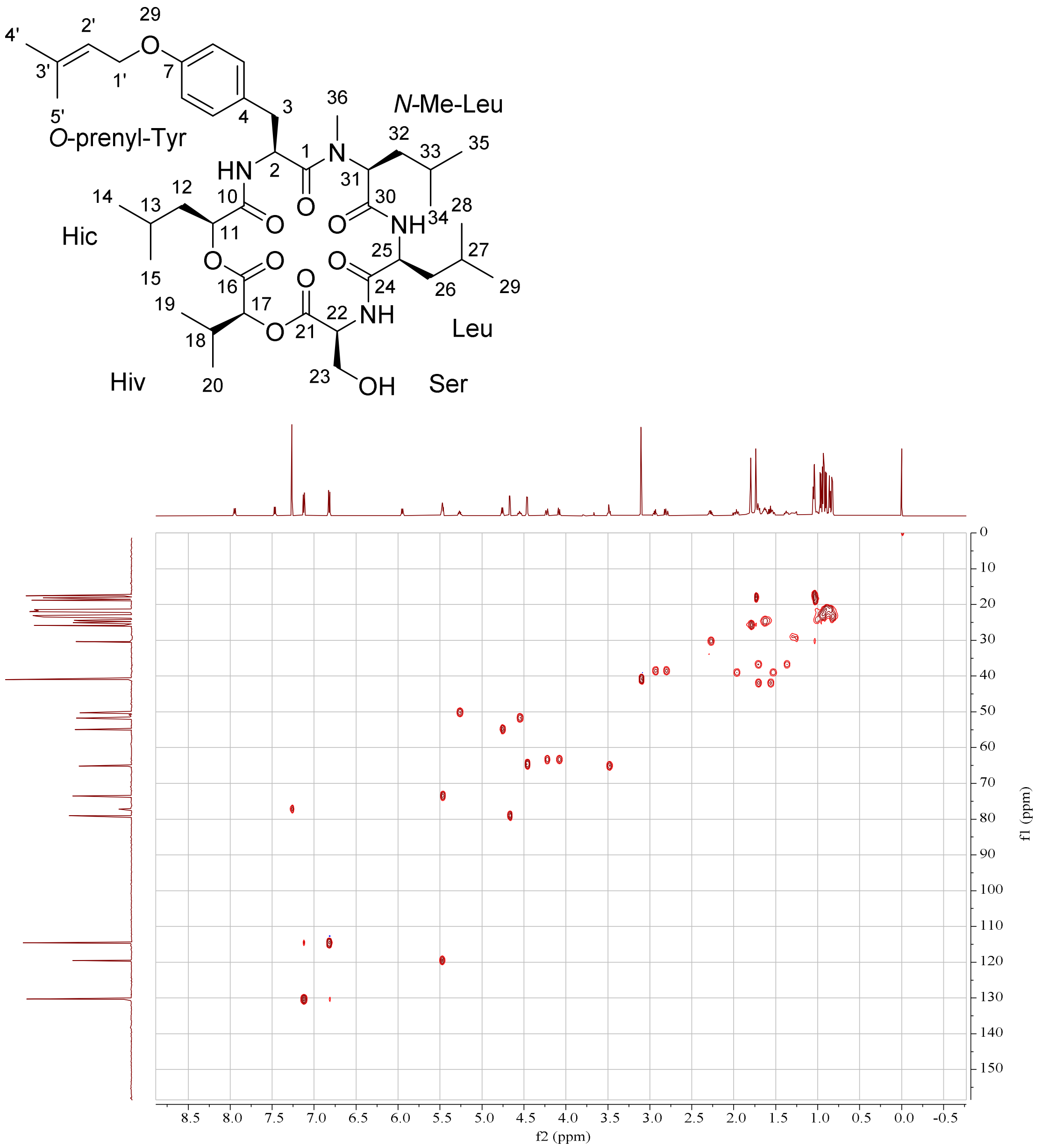

Figure S6. HSQC spectrum of **1** in $CDCl_3$.

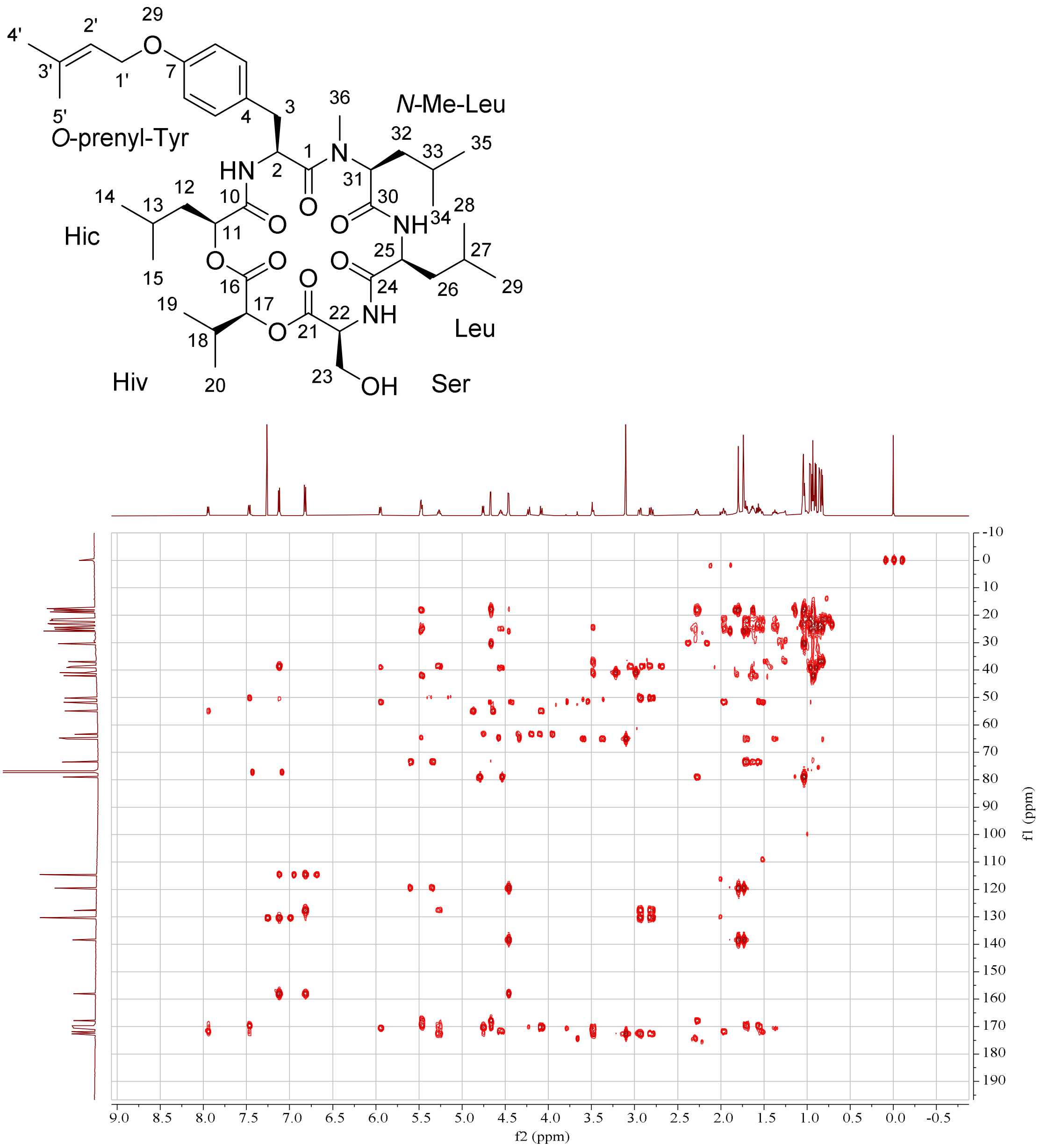

Figure S7. HMBC spectrum of **1** in $CDCl_3$.

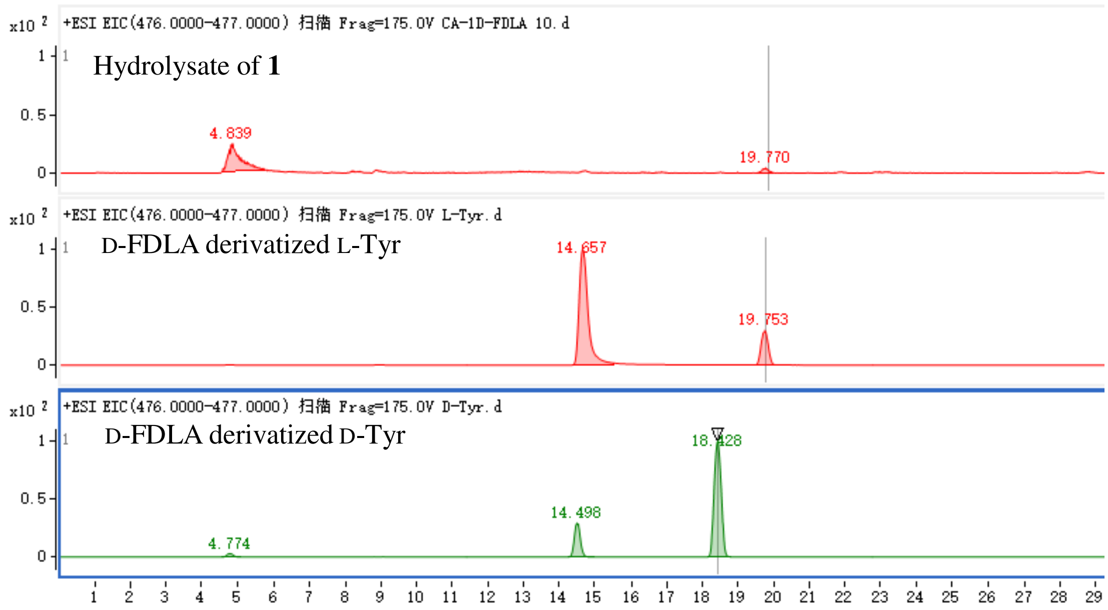
+ESI EIC(476.0000-477.0000) 扫描 Frag=175.0V CA-1D-FDLA 10.d
Hydrolysate of 1
4.839
19.770
+ESI EIC(476.0000-477.0000) 扫描 Frag=175.0V L-Tyr.d
D-FDLA derivatized L-Tyr
14.657
19.753
+ESI EIC(476.0000-477.0000) 扫描 Frag=175.0V D-Tyr.d
D-FDLA derivatized D-Tyr
18.428
14.498
4.774

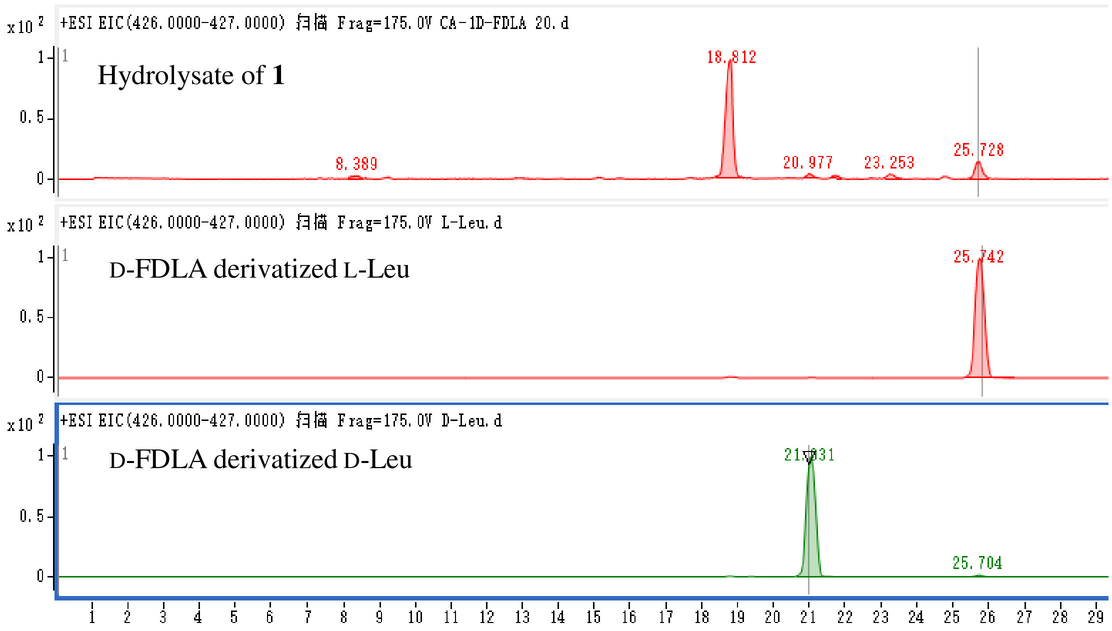
+ESI EIC(426.0000-427.0000) 扫描 Frag=175.0V CA-1D-FDLA 20.d
Hydrolysate of 1
18.812
8.389
20.977
23.253
25.728
+ESI EIC(426.0000-427.0000) 扫描 Frag=175.0V L-Leu.d
D-FDLA derivatized L-Leu
25.742
+ESI EIC(426.0000-427.0000) 扫描 Frag=175.0V D-Leu.d
D-FDLA derivatized D-Leu
21.031
25.704

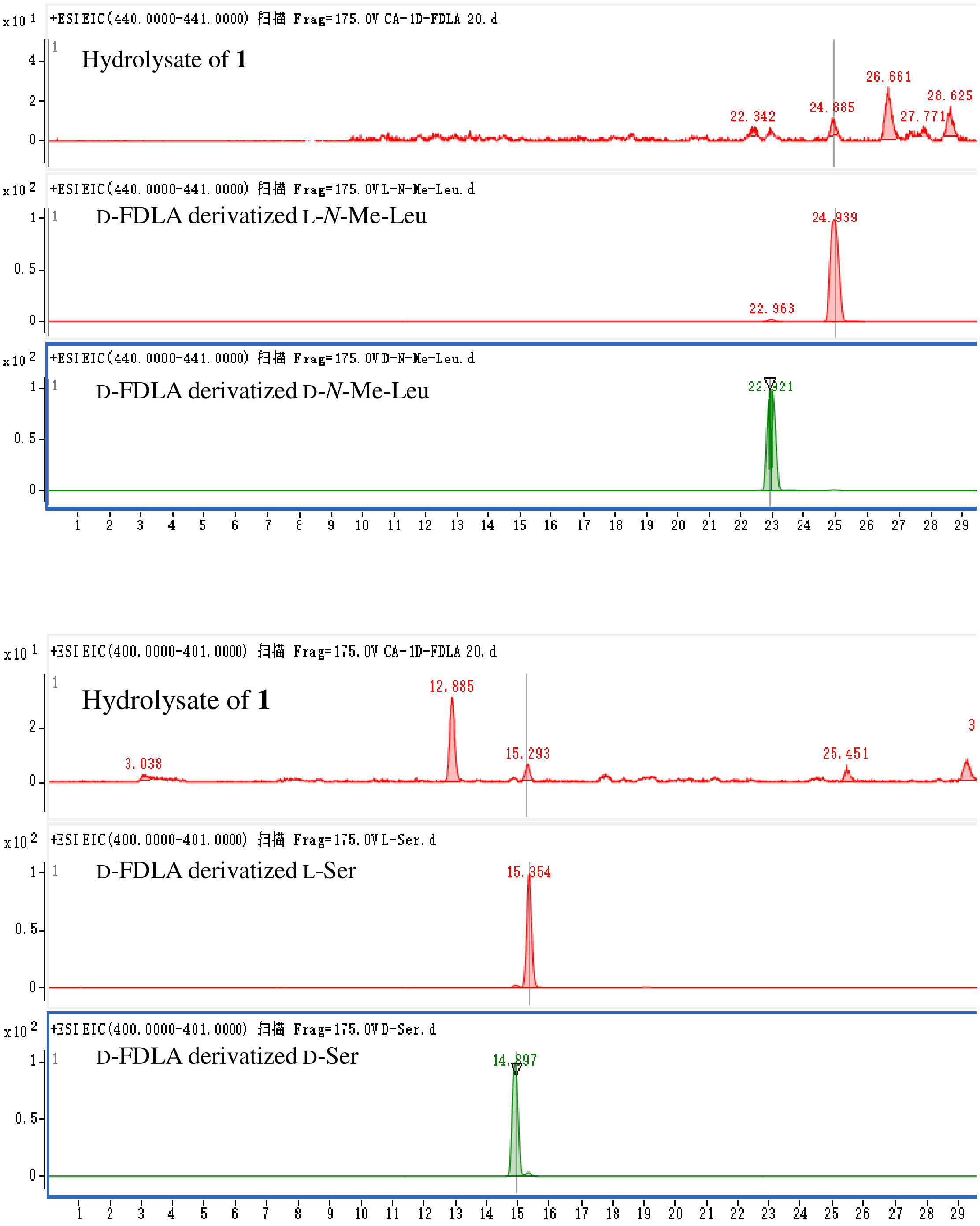

Figure S8. Chromatographic profiles of amino acids from compound **1** acid hydrolysis products

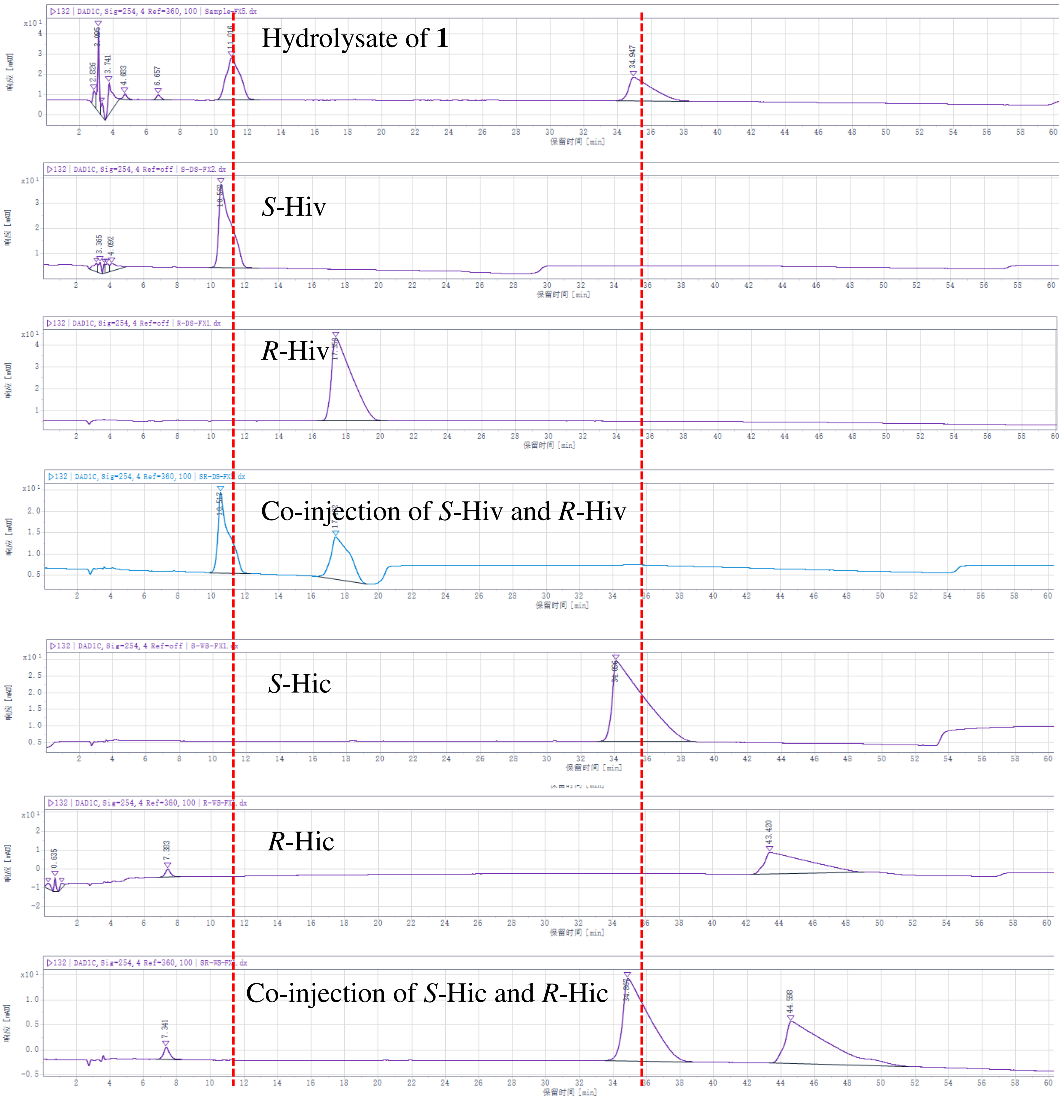

Figure S9. Chiral HPLC analysis performed on hydrolysis products of **1**.
